# A Two-Dimensional Model of Potassium Signaling and Oscillatory Growth in a Biofilm

**DOI:** 10.1007/s11538-021-00887-3

**Published:** 2021-04-15

**Authors:** Noah Ford, Garth Fisher, Arthur Prindle, David Chopp

**Affiliations:** 1 Engineering Sciences and Applied Mathematics Department, Northwestern University, Evanston, IL 60208 USA; 2 Department of Biochemistry and Molecular Genetics, Feinberg School of Medicine, Northwestern University, Chicago, IL 60611 USA; 3 Center for Synthetic Biology, Northwestern University, Evanston, IL 60208 USA; 4 Department of Chemical and Biological Engineering, Northwestern University, Evanston, IL 60208 USA

**Keywords:** Biofilms, Mathematical modeling, Oscillatory behavior

## Abstract

Biofilms are complex communities of bacteria that exhibit a variety of collective behaviors. These behaviors improve their ability to survive in many different environments. One of these collective behaviors seen in *Bacillus subtilis* is the ability for starving cells to stop the growth of other cells using potassium signaling and voltage changes. This signaling produces an oscillatory growth pattern so that during periods of low growth the nutrients diffuse deeper into the biofilm and reach the nutrient-starved, interior regions of the biomass. In this paper, we develop a mathematical model to describe this oscillatory behavior, and we use this model to develop a two-dimensional simulation that reproduces many of the important features seen in the experimental data. This simulation allows us to examine the spatial patterning of the oscillatory behavior to better understand the relationships between the various regions of the biofilm. Studying the spatial components of the metabolic and voltage oscillations could allow for the development of new control techniques for biofilms with complex shapes.

**Supplementary Information:**

The online version contains supplementary material available at 10.1007/s11538-021-00887-3.

## Introduction

Biofilms are collections of bacteria that grow on surfaces, which can significantly impact engineered systems and human health. For example, biofilms play a role in many infections in humans (Costerton et al. (1999); Parsek and Singh (2003)). Biofilms exhibit several unique, collective behaviors that improve the bacteria’s ability to survive in a variety of environments. Some known, collective behaviors include symbiotic relationships between multiple species within a biofilm and the development of physical channels between cells to facilitate nutrient penetration and distribution. More information on these well-studied, collective behaviors can be found in Costerton (2007). In this paper, we explore an oscillatory growth pattern that emerges from potassium signaling in some biofilms of *Bacillus subtilis*. These oscillations were first reported in Jintao et al. (2015) and in Prindle et al. (2015) as another mechanism for cooperation within a biofilm that may improve their ability to survive and grow in certain environments. By studying this oscillatory mechanism, we may be able to better understand certain pathogenic biofilm infections and find new ways to fight them.

The bacteria in these biofilms depend on limited nutrients from the fluid in order to survive. As a biofilm grows larger, cells near the interior often become starved for nutrients that must travel farther through active biomass to reach them. If the cells near the periphery of the biofilm continue to grow at the same rate, the cells near the center of the biofilm could die from starvation. A high level of cell death near the center of a biofilm could destabilize the biofilm and adversely affect the peripheral cells’ survival. This conflict between the growth of cells near the biofilm interface and the maintenance of the interior cells is discussed in Jintao et al. (2015). In the experiments in Prindle et al. (2015) the primary nutrient is glutamate, which is a nitrogen source that cells use for both growth and maintenance. As shown in Prindle et al. (2015), the electrochemical signaling is driven by the cellular release and uptake of potassium, a positive ion that the cells use to regulate their voltage differential. When cells become metabolically stressed, they release potassium and hyperpolarize as shown in Prindle et al. (2015). This release of potassium causes neighboring cells to uptake potassium and briefly depolarize, which interferes with their metabolic processes. Once these neighboring cells become stressed, they also release potassium and hyperpolarize, as shown experimentally in Prindle et al. (2015). Collectively, the cells generate a potassium wave that travels from the nutrient-starved interior to the exterior of the biofilm. This wave disrupts the entire biofilm’s nutrient consumption. The disruption in consumption allows nutrients to diffuse past the periphery toward the starving interior cells allowing the biofilm to maintain a moderated growth rate while avoiding a destabilizing level of cell death in its interior, as explained in Jintao et al. (2015).

Previous work using one-dimensional models also suggests that the oscillations arise from metabolic stress and propagate through changes in potassium, as demonstrated by the model introduced in Martinez-Corral et al. (2019). In this paper, we build on this metabolic propagation mechanism by introducing a new method of cell-to-cell communication in which the cells react to changing potassium levels instead of the absolute potassium level. This model is consistent with the observation that bacteria can adjust to living in environments that possess a range of potassium concentrations. *B. subtilis* appears to use multiple types of transporters to maintain a homeostatic, internal potassium concentration, and the expression of each transporter allows the bacteria to grow at different potassium concentrations ( Gundlach et al. (2017)). While we do not have data that quantifies the cells’ ability to adjust to various potassium concentrations, we fit by hand a linear differential equation that represents this assumption. We use this set of equations to develop a two-dimensional model to more accurately represent the experiments in Prindle et al. (2015) and to study variations in the oscillation patterns seen in these experiments. This model is useful in studying multi-dimensional phenomena that appear within biofilm systems such as the communication between separated biofilms.

## One-Dimensional Model

We first develop a one-dimensional model that represents a cross section of the biofilm and is based on the continuum model developed in Wanner and Gujer (1986). The model relies on cellular metabolism to initiate and propagate the signal. These oscillations have been linked to metabolism in previous research, such as in Jintao et al. (2015) and in Liu et al. (2017). A biofilm begins to oscillate at a smaller size in environments with lower ambient glutamate as shown in Martinez-Corral et al. (2019). This result suggests that there is a glutamate threshold under which the cells become stressed and hyperpolarize. Metabolism also plays an important role in the one- and two-dimensional model we develop in this paper.

In developing our model, we consider a model proposed by Martinez-Corral et al. (2019) that is able to replicate much of the behavior that we see in experiments. The main differences between the model from Martinez-Corral et al. (2019) and the model introduced here is that we change the cells’ response to extracellular potassium, we simplify the boundary condition, and we more closely tie together the equations for voltage and potassium, specifically for the processes of potassium leakage and potassium uptake. We find that this updated model better captures the inverse relationship between the voltage and the extracellular potassium seen in experiments from Prindle et al. (2015).

We first consider how a rise in the external potassium concentration can affect the metabolism of a cell. We assume that a rise in environmental potassium causes potassium to leak into the bacteria, thereby depolarizing the cell and affecting its glutamate consumption. While there are a few ways through which voltage changes can affect consumption, Martinez-Corral et al. (2019) assume that a bacterium cannot uptake glutamate if it is depolarized away from its homeostatic voltage even if glutamate is environmentally available. It is possible that the voltage change affects other cellular processes and molecules such as ammonium within the cell instead of the glutamate intake, which would in turn affect the usage of glutamate. The role of ammonium in these oscillations is examined in Jintao et al. (2015). A model based on changes to other metabolic processes would likely lead to a similar decrease in the cellular metabolism of depolarized cells as the model that is based on a diminished glutamate uptake. While the biological processes that lead to the stressed response are worthy of further study, we cannot fully address them in this modeling study. The important effect for the purposes of this model is that depolarized cells metabolize glutamate more slowly, and they become stressed and hyperpolarize to recover. The mechanism for the reduced glutamate metabolism that we use in this model is the same as that used in Martinez-Corral et al. (2019), which assumes that depolarized cells cannot uptake glutamate.

In our model, we introduce a diffusive-flux boundary condition at the biofilm interface. The model in Martinez-Corral et al. (2019) uses an artificial flux approximation to calculate the glutamate and potassium influx. It defines the exchange of these molecules at a point within the biofilm as a function of that point’s distance to the exterior interface. In this model, we replace this boundary calculation with a traditional Neumann flux boundary condition because the Neumann boundary condition is simpler to implement for two-dimensional biofilms with irregular shapes.

Potassium, a positively charged ion, is the main signaling molecule in this model, and it diffuses in the spaces between the cells. Because we do not have experimental results that allow us to directly measure this diffusion process, we fit the model’s potassium diffusion rate by comparing its extracellular potassium concentration to the potassium concentration observed in experiments. In the experiments, we see high concentrations of potassium throughout the biofilm for an extended period after the cells hyperpolarize. Many biofilm models assume that the diffusion rate inside the biofilm is around $60\%$ of the diffusion rate in the fluid. Testing this assumption, we find that a model with a potassium diffusion rate in the biofilm that is less than $60\%$ of the fluid diffusion rate better matches the higher experimental potassium concentrations. A diffusion rate of $60\%$ leads the potassium to diffuse too quickly out of the biofilm and into the flow. Our usage of a lower diffusion rate agrees with the results from Larkin et al. (2018) in which the potassium diffusion is modeled as a percolation process where potassium released from one cell can only affect the cell’s direct neighbors. The authors find that the percentage of cells that participate in the voltage oscillations reflects the percentage required for efficient transmission in percolation theory. This result suggests that potassium cannot diffuse far from the cell from which it originates, supporting our decision to model this confined movement by setting the diffusion coefficient for potassium within the biofilm to be significantly smaller than the coefficient within the fluid.

The diffusion of glutamate and potassium ions through the charged biomass is a complicated process, and it merits further study outside the scope of this project. Instead of assuming that the diffusion rate inside the biofilm is some fitted fraction of the diffusion rate in fluid, one can calculate a diffusion rate that takes into account the shape of the bacteria with a technique used in Fort et al. (2002). The interactions of glutamate and potassium’s charges may also be important for calculating their diffusion. We examine our assumption to approximate the diffusion rate in the following section.

The equations for the external glutamate and potassium concentrations, *G* and *K*, respectively, within the biofilm are defined below:

1 $$\begin{aligned} \frac{\partial G}{\partial t}&= D_G\frac{\partial ^2G}{\partial x^2}-\frac{\delta _{G}}{(1+\exp (V-V_{th}))}G(G_{max}-G_{in}), \end{aligned}$$

2 $$\begin{aligned} \frac{\partial K}{\partial t}&= D_K\frac{\partial ^2K}{\partial x^2}+Fg_Kn^4(V-V_K)+Fg_L(V-V_L) \nonumber \\&- \max (\gamma _KK(K_{max}-K_{in}),0). \end{aligned}$$

Glutamate and potassium diffuse through the biofilm in the spaces between the cells, and this diffusion is represented by the first terms of Equations (1) and (2). Their diffusion rates are defined as $D_G$ and $D_K$, respectively. The values used for these and other parameters in the simulations are listed in Table 1.

**Table 1:** Parameters Used in Simulation

Name	Description	Value	Reference
$D_G$	Glutamate diffusion coefficient in biofilm	0.540 $\frac{\text{ mm}^2}{\text{ day }}$	Assumed
$D_K$	Potassium diffusion coefficient in biofilm	0.497 $\frac{\text{ mm}^2}{\text{ day }}$	Fitted
$D_G^{fl}$	Glutamate diffusion coefficient in fluid	0.900 $\frac{\text{ mm}^2}{\text{ day }}$	Known
$D_K^{fl}$	Potassium diffusion coefficient in fluid	4.97 $\frac{\text{ mm}^2}{\text{ day }}$	Known
$G_0$	Glutamate concentration at inlet	30 mM	Prindle et al. (2015)
$K_0$	Potassium concentration at inlet	8 mM	Prindle et al. (2015)
$\delta _G$	Glutamate uptake rate	10 $\frac{1}{\text{ hour }}$	Fitted
$V_{th}$	Voltage above which cells cannot uptake glutamate	$-150$ mV	Prindle et al. (2015)
$G_{max}$	Maximum glutamate concentration in cell	20 mM	Fitted
*F*	Voltage to potassium conversion factor	$5.6\frac{\text{ mM } \text{ K }}{\text{ mV }}$	Prindle et al. (2015)
$g_K$	Potassium gate strength	180/hour	Fitted
$g_L$	Leak gate strength	1.2/hour	Fitted
$\gamma _K$	Potassium pump strength	$.025/(\text{ hour }\times \text{ mM } \text{ K})$	Fitted
$B_L$	Boundary layer length	0.5 mm	Fitted
$\gamma _G$	Glutamate consumption rate	$1.125/\text{hour }$	Fitted
$r_b$	Glutamate basal consumptions rate	0.1 (dimensionless)	Fitted
$G_u$	Glutamate bound below which the cells do not grow	18 mM Glutamate	Fitted
$\eta _V$	Voltage influence in $M_{grow}$	20 (dimensionless)	Fitted
$\gamma _V$	Voltage transition speed in $M_{grow}$	20 (dimensionless)	Fitted
$V_{low}$	Voltage below which cells do not grow	$-175$ mV	Fitted
$\delta _{grow}$	Biomass produced in growth	$\dfrac{0.0075\text{ mm }}{\text{ mM } \text{ Glutamate }\times \text{ hour }}$	Fitted
$\eta _K$	Cells’ acclimation rate to potassium change	30/hour	Fitted
$\alpha$	Potassium gate opening rate	5/hour	Fitted
$\beta$	Potassium gate closing rate	2.5/hour	Fitted
*m*	Potassium gate exponent	2 (dimensionless)	Fitted
$G_l$	Glutamate level below which cells hyperpolarize	10 mM Glutamate	Fitted
$V_{K0}$	Basal potassium gate reversal potential	$-380$ mV	Prindle et al. (2015)
$V_{L0}$	Basal leak gate reversal potential	$-156$ mV	Prindle et al. (2015)
$\delta _K$	Potassium gate reversal change	$1 \frac{\text{ mV }}{\text{ mM }}$	Prindle et al. (2015)
$\delta _L$	Leak gate reversal change	$60 \frac{\text{ mV }}{\text{ mM }}$	Fitted
$\alpha _T$	ThT fluorescence strength	20 $\frac{\text{ mM }}{\text{ hour }}$	Fitted
$g_T$	ThT relation to voltage	0.3 mV	Martinez-Corral et al. (2019)
$V_{0T}$	Voltage level below which ThT fluoresces	$-170$ mV	Fitted
$\gamma _T$	ThT decay rate	10/hour	Martinez-Corral et al. (2019)
$\alpha _A$	APG fluorescence strength	0.5/hour	Fitted
$\gamma _A$	APG decay rate	1/hour	Fitted

The second term in Equation (1) models the glutamate uptake by the bacteria. The bacteria uptake glutamate if they are sufficiently polarized, there is glutamate in the environment, and the cells’ internal glutamate level is below their maximum concentration. The cells uptake glutamate using transporters powered by the proton motive force, which is explained in Martinez-Corral et al. (2019). The cells must maintain a certain level of polarization to uptake glutamate, which is examined in Tolner et al. (1995). We use the same function for the dependence of glutamate uptake on voltage as Martinez-Corral et al. (2019). The exponential term in this expression corresponds to the quick halting of glutamate uptake if the cells’ voltage differential, *V*, moves above their homeostatic voltage differential, $V_{th}$, thereby decreasing the magnitude of their voltage differential. This exponential relationship assumes that the glutamate transporters are very sensitive to voltage changes. Glutamate uptake is also dependent on the glutamate availability in the environment and on the cells’ need for glutamate, which we define as the difference between the maximum interior glutamate level, $G_{max}$, and the cells’ internal glutamate level, $G_{in}$.

Equation (2) represents the external potassium concentration as the molecules diffuse through the biofilm and move through the cellular membrane both passively and actively. Potassium moves passively through the potassium gates and through the leak gates. The potassium gates are channels that the cell can open and close to allow potassium to enter or leave the cell. The leak gates represent the permeability of the cell membrane, which allows potassium to enter or leave the cell through small holes. In the model, the potassium and leak gate are controlled by the openness of the potassium gates, *n*, and the corresponding reversal potentials for the potassium and leak gates, $V_K$ and $V_L$. Here, *F* is a factor converting the voltage change to a potassium change. We approximate the potassium uptake and release through the potassium and leak gates using terms from the Hodgkin–Huxley Model introduced in Hodgkin and Huxley (1952). Allowing the extracellular potassium to move into the cells through the leak gates and decrease the external potassium concentration is a new feature of this model that reflects the mechanism through which the extracellular potassium depolarizes a cell. A higher concentration of extracellular potassium increases the potassium uptake due to an increased osmotic pressure. Previous models focused on how this potassium movement through the leak gates affects the voltage differential but did not account for the effects within the potassium concentration itself. Including this uptake in the model ensures that the increase in extracellular potassium during a depolarization event is moderated by the potassium uptake of the depolarizing cells. The final term in Equation (2) represents the cells’ potassium pumps through which they can actively uptake nearby potassium if their internal potassium concentration, $K_{in}$, falls below a threshold, $K_{max}$. The max function in this potassium pump term ensures that the cells only use this pathway to uptake potassium and not to release potassium. This simplified representation of potassium uptake does not take into account the internal glutamate concentration, which has been found to affect the potassium uptake of at least one potassium channel, KtrCD. A higher glutamate concentration increases the potassium channel KtrCD uptake of potassium at low external potassium concentrations ( Krüger et al. (2020)). We leave modeling the interaction of glutamate concentration and potassium uptake to future work.

The boundary conditions for glutamate and potassium at the biofilm’s exterior interface require that the concentrations and fluxes are continuous across the boundary, which are described by the Neumann flux conditions below:

3 $$\begin{aligned}&D_G\frac{\partial G}{\partial x}&=D_G^{fl}(G_{0}-G_{int})/B_L, \end{aligned}$$

4 $$\begin{aligned}&\quad D_K\frac{\partial K}{\partial x}&= D_K^{fl}(K_{0}-K_{int})/B_L, \end{aligned}$$

where $D_G^{fl}$ and $D_K^{fl}$ are the diffusion rates of glutamate and potassium in the fluid, $G_{0}$ and $K_{0}$ are the long-range glutamate and potassium concentrations, $G_{int}$ and $K_{int}$ are the interfacial glutamate and potassium concentrations, and $B_L$ is the boundary layer width. At $x=0$, we represent the interior wall with the no-flux boundary conditions of $\partial G/ \partial x = \partial K / \partial x= 0$.

The corresponding equations for the glutamate and potassium concentrations in the interior of the cell, $G_{in}$ and $K_{in}$, respectively, are as follows:

5 $$\begin{aligned} \frac{dG_{in}}{dt}&= \frac{\delta _{G}}{(1+\exp (V-V_{th}))}G(G_{max}-G_{in}) - \gamma _{G}G_{in}(M_{grow}+r_b)\nonumber \\&\quad - \frac{\partial }{\partial x} (U G_{in}), \end{aligned}$$

6 $$\begin{aligned} \frac{d K_{in}}{dt}&= -Fg_Kn^4(V-V_K)-Fg_L(V-V_L) + \max (\gamma _KK(K_{max}-K_{in}),0)\nonumber \\&\quad - \frac{\partial }{\partial x} (U K_{in}). \end{aligned}$$

Equation (5) models the glutamate concentration inside the bacteria. The first term represents the cells’ glutamate intake. This term balances the external glutamate concentration in Eq. (1), but with an opposite sign to represent the transport of glutamate across the cell membrane. The second term represents the cells’ glutamate consumption for both its base metabolism and growth, where $\gamma _{G}$ is the glutamate consumption rate. The cells require glutamate to perform their base metabolic functions, and this need is represented as $r_b$ in the equation.

The cells also use glutamate to grow, and their growth propensity is represented by the variable $M_{grow}$. Growth can only occur when the bacteria are in “Grow Mode,” or when $M_{grow}$ is high. When the bacteria are stressed, $M_{grow}$ is low, which slows the glutamate consumption of the bacteria and allows for the glutamate to penetrate the biofilm more deeply. We discuss this variable in more detail below. The model in Martinez-Corral et al. (2019) also includes a term through which the cells regulate their growth in response to their changing environment. In their model, the cells uptake more glutamate when they have a higher internal glutamate level. This property creates a delay in the glutamate uptake and growth that prevents the bacteria from reaching a steady state, which allows for sustained oscillations. In this model, we use $M_{grow}$ as a variant of this consumption delay where the biofilm cannot be hyperpolarized and grow.

Equation (6) represents the cells’ internal potassium concentration. The first three terms of this equation are also found in Eq. (2) to represent the transport of potassium across the cell membrane.

The advection terms within Eqs. (5) and (6) reflect that $G_{in}$ and $K_{in}$ are quantities within an individual cell that are pushed outward as the biofilm grows. Here, *U* is the biofilm growth velocity defined throughout the biofilm. The growth velocity at each point in a one-dimensional cross section of a biofilm is the sum of the biomass growth between that point and the immobile wall. As bacteria reproduce, they push the biomass away from the wall and farther into the fluid flow, which is discussed in Wanner and Gujer (1986). All non-diffusive quantities in this system move with the cellular growth and have a corresponding advection term in their equations.

The following equations define $M_{grow}$ and the corresponding growth equations:

7 $$\begin{aligned} M_{grow}&= \frac{T_G}{T_G + T_V} \nonumber \\&=\frac{G_{in}}{G_{in}+G_u}/\left( \frac{G_{in}}{(G_{in}+G_u)}+(\eta _V\tanh (\gamma _V(V/V_{low}-1))+1)\right) , \end{aligned}$$

8 $$\begin{aligned} U(x)&= \delta _{grow}\int _0^x G_{in}M_{grow}dx, \end{aligned}$$

9 $$\begin{aligned} \frac{dL}{dt}&= \delta _{grow}\int _0^L G_{in}M_{grow}dx. \end{aligned}$$

Equation (7) defines the variable $M_{grow}$, which reflects the growth propensity of the cells. The variable $M_{grow}$ varies between zero and one where the cells grow faster if $M_{grow}$ is close to one. Let $T_G = \frac{G_{in}}{G_{in}+G_u}$ and $T_V = \left( \eta _V\tanh (\gamma _V(V/V_{low}-1))+1\right)$. Then, $T_G$ is a Hill activation function that is large when $G_{in}$ is higher than the lower bound $G_u$, and $T_V$ is a hyperbolic tangent activation function that is large when *V* is above the bound $V_{low}$. The parameters $\eta _V$ and $\gamma _V$ are shape parameters for the hyperbolic tangent activation function. Equation (7) is the steady-state solution of the differential equation $\frac{\partial M_{grow}}{\partial t} = (1-M_{grow}) T_G - M_{grow} T_V$. Then, $M_{grow}$ will be close to 1 and the bacteria will grow if $T_G$ is much larger than $T_V$. The variable $M_{grow}$ ensures that the cells only grow when both their internal glutamate is high, making $T_G$ large, and they are not hyperpolarized, making $T_V$ small. Since cells consume glutamate at a much lower rate when hyperpolarized, the nutrients can penetrate deeper into the biofilm and arrive at the starving cells near the interior. The requirement that the cells be near their homeostatic voltage differential in order to grow reflects what we see in experimental data from Larkin et al. (2018).

As developed in Wanner and Gujer (1986), Equations (8) and (9) represent the biofilm’s growth, where $\delta _{grow}$ is the growth rate. Equation (8) computes the growth at any point within the biofilm as an integral of the biomass growth between that point and the wall. Equation (9) represents the growth of the biofilm’s length, *L*, which is the distance between the base and the edge of the biofilm.

**Fig. 1:**
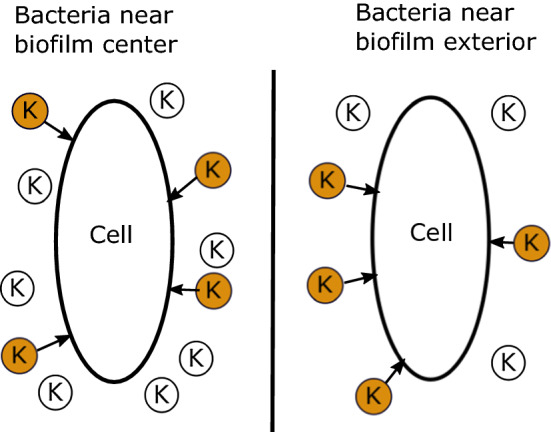
The effects of extracellular potassium on cells near the center of the biofilm versus cells near the exterior. The potassium ions in orange are newly arrived molecules, and we assume that they have same depolarizing effect on both cells despite differing ambient potassium concentrations (Color figure online)

The most important difference in this model compared to previous ones relates to how the cells react to potassium. In our model, cells respond to changes in potassium instead of the absolute potassium level as represented in Fig. 1. We assume that only newly arriving potassium molecules affect the cells’ voltages. We base this assumption on the evidence that bacteria can adjust to a range of potassium levels over time. Three of the mechanisms that cells use to regulate potassium are studied in Gundlach et al. (2017). The authors show that cells that express only one of the three transporters, KtrAB, KtrCD, and KimA, reach their half-maximal growth rates at different external potassium concentrations, suggesting that a cell’s ability to uptake potassium through its various transporters impacts its growth. In this model, we assume that a change in external potassium temporarily affects the cell’s ability to maintain its homeostatic voltage, and the voltage change disrupts the cell’s growth. A change in environmental potassium could be a better indicator of cellular stress than the absolute potassium level because a change in potassium could force bacteria out of their equilibrium state. It is likely that the absolute potassium level, as well as other factors, plays a role in the cells’ glutamate uptake, but in this model, we consider a change in potassium as the main influencing force. We create the variable $K_{acclimated}$ to represent the potassium level to which the cells are accustomed. This variable then follows *K* linearly at a rate of $\eta _K$ in the following equation:

10 $$\begin{aligned} \frac{d K_{acclimated}}{dt}&= \eta _K(K-K_{acclimated}) - \frac{\partial }{\partial x}(UK_{acclimated}). \end{aligned}$$

The voltage is affected by the difference between the environmental potassium level, *K*, and the level to which the cell is accustomed, $K_{acclimated}$, which is incorporated into the reversal potential for the leak gates $V_L$. The following set of equations describe the voltage differential within the biofilm:

11 $$\begin{aligned} \frac{dV}{dt}&= -g_kn^4(V-V_K) - g_L(V-V_L)\nonumber \\&\quad -\max (\gamma _KK(K_{max}-K_{in}),0)/F - \frac{\partial }{\partial x} (U V), \end{aligned}$$

12 $$\begin{aligned} \frac{dn}{dt}&= \alpha \frac{(G_{max}-G_{in})^m}{(G_{max}-G_{l})^m+(G_{max}-G_{in})^m}(1-n) - \beta n - \frac{\partial }{\partial x} (U n), \end{aligned}$$

13 $$\begin{aligned} V_K&= V_{K0} + \delta _KK, \end{aligned}$$

14 $$\begin{aligned} V_L&= V_{L0} + \delta _L(K-K_{acclimated}). \end{aligned}$$

Equation (11) represents the voltage differential across the cells’ membranes, *V*. In this model, the cells use potassium to modify this voltage differential. The terms in this equation are from the Hodgkin–Huxley model, and they correspond to the potassium and leak gate terms from the potassium equations, Eqs. (2) and (6). In this model, we assume that the only molecule moving through the leak gates is potassium, and we do not include the other ions that likely move through the leak gate. We observed oscillations by solely considering the effects of potassium on the cell, but future work should examine the potential role of other ions within the cells’ polarization processes. The penultimate term in Eq. (11) corresponds to the cells’ active pumping of potassium to maintain their voltage differential, and it is normalized here by *F*. The model does not account for changes in the voltage differential caused by diffusing, external potassium, which might also be important to the oscillations.

Equation (12) represents the openness of the cells’ potassium gates, *n*. The gates open with the opening rate $\alpha$ according to the expression

$$\begin{aligned}\alpha \frac{(G_{max}-G_{in})^m}{(G_{max}-G_{l})^m+(G_{max}-G_{in})^m},\end{aligned}$$

which is similar to a Hill function with *m* as the Hill coefficient. However, this expression is large when $G_{in}<G_l$ and small when $G_{in}>G_l$. This expression activates the starving response in the bacteria when their internal glutamate level is low. With low internal glutamate, the bacteria open their potassium gates to release potassium and hyperpolarize. The second term in Eq. (12) represents that bacteria close their potassium gates at a decay rate of $\beta$ if they are not experiencing stress.

The reversal potentials $V_K$ and $V_L$ are defined in Eqs. (13) and (14). The reversal potential for the potassium gates, defined in Equation (13), has a base value of $V_{K0}$ and is affected by the external potassium concentration with an influence strength of $\delta _K$. The model in Martinez-Corral et al. (2019) uses a Nernst potential to define the potassium reversal potential. While we recognize the merit of this approach, we simplify the equation here to be of the same form as the corresponding equation for the leak gates. The reversal potential for the leak gates, defined in Eq. (14), has a base value of $V_{L0}$ and is affected by the difference between the environmental potassium level, *K*, and the level to which the cell is accustomed, $K_{acclimated}$. The parameter $\delta _L$ defines the strength of this influence. A rise in the external potassium leads the potassium to leak into the bacteria causing the cells to depolarize.

Most experimental data from these systems are reported as the fluorescence of molecular indicators. To enable comparison, we convert both the voltage and potassium concentrations to the fluorescent intensity of their corresponding indicators, thioflavin T (ThT) and Asante potassium green (APG), named *T* and *A*, respectively, in the equations below:

15 $$\begin{aligned} \frac{dT}{dt}&= \frac{\alpha _T}{1+\exp (g_T(V-V_{0T}))} - \gamma _T T - \frac{\partial }{\partial x} (U T), \end{aligned}$$

16 $$\begin{aligned} \frac{dA}{dt}&= \alpha _AK - \gamma _A A - \frac{\partial }{\partial x} (U A). \end{aligned}$$

Equation (15) has a similar form to the ThT fluorescence equation used in Martinez-Corral et al. (2019). The indicator ThT fluoresces if the voltage falls below the threshold $V_{0T}$. The exponential term produces a strong transition point for ThT fluorescence if the voltage differential drops below $V_{0T}$. The parameter $g_T$ adjusts ThT’s sensitivity to this transition point, and $\gamma _T$ is the decay rate of the indicator. Equation (16) uses a linear model to represent the fluorescence of APG where $\alpha _A$ is the activation strength of APG, and $\gamma _A$ is the decay rate of the indicator.

Together the equations presented in this section form an oscillatory system for both the growth and the voltage differential. The starving cells release potassium, which disrupts the metabolism of neighboring cells, causing them to become stressed and stop growing. The cells release potassium to hyperpolarize when stressed which creates the potassium wave that moves from the center of the biofilm to the exterior. The hyperpolarized bacteria refrain from growing while recovering, allowing glutamate to diffuse deeper into the biofilm. This one-dimensional model reproduces prominent features from experiments as discussed in the following sections.

### Comparing Voltage, Growth, and Potassium to Experiments

**Fig. 2:**
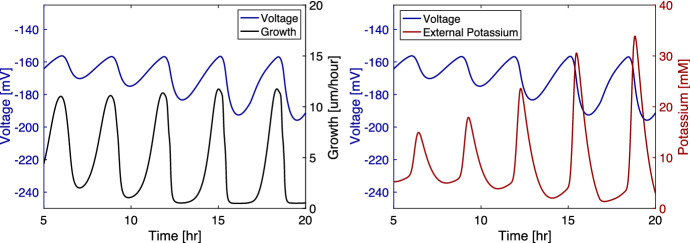
(Left) Mean voltage and growth over time from the model. Voltage is positively related to growth. The oscillations become stronger over time. Highest growth occurs when the biofilm is not hyperpolarized. (Right) Mean voltage and mean potassium over time from our model. Voltage is negatively related to potassium. Potassium is at its highest when the biofilm is depolarizing as the cells release potassium. Potassium begins to fall before the voltage increases because some of the potassium diffuses into the bulk flow (Color figure online)

**Fig. 3:**
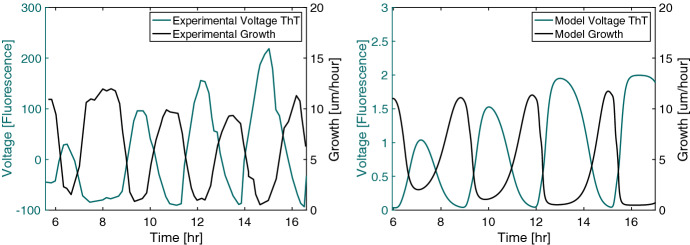
(Left) Experimental mean voltage fluorescence and growth adapted from Prindle et al. (2015). Voltage is measured as its fluorescent indicator, ThT, which exhibits higher fluorescence when the cell is more polarized meaning that the voltage differential is more negative. The voltage fluorescence and the growth are inversely related. (Right) Mean voltage fluorescence and growth from the model. The quantities from the model demonstrate a similar pattern to those from the experiment. Note that the fluorescence data from the experiment are scaled differently than the model data (Color figure online)

To validate the set of equations introduced in the previous section, we compare important quantities from the model to the experimental data such as the relationship between voltage oscillations, the growth, and the potassium concentration. We first examine how hyperpolarization affects a cell’s growth rate. Experiments show that a biofilm typically does not grow when its cells are hyperpolarized ( Larkin et al. (2018)). Our model prevents bacteria from growing while hyperpolarized through the $M_{grow}$ variable. This effect can be seen in Fig. 2, which shows that the biofilm grows faster when its average voltage is higher. The biofilm’s initial width is 150 microns. We compare this relationship to experimental data from Prindle et al. (2015) in Fig. 3 in which the biofilm’s initial values for the data fields are the same as in the previous simulation. We see in both the experimental data and the simulation that growth is high when the ThT fluorescence (which indicates hyperpolarization) is low, and the growth is low when the fluorescence is high.

**Fig. 4:**
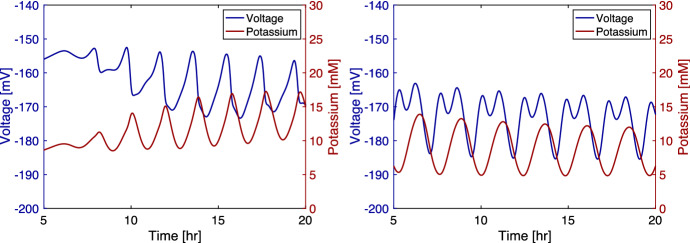
(Left) Mean voltage and potassium from an implementation of the model from Martinez-Corral et al. (2019). In this model, voltage and potassium appear to be inversely related. (Right) Mean voltage and potassium from an implementation of the same model but with a Neumann flux boundary condition and a constant internal diffusion rate. Here, we see that extracellular potassium is increasing while mean voltage initially rises (Color figure online)

**Fig. 5:**
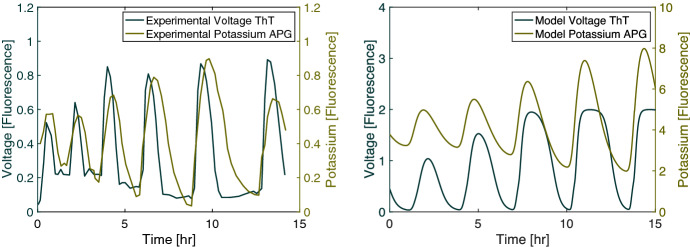
(Left) Experimental mean voltage and potassium fluorescence adapted from Prindle et al. (2015). The fluorescence of the voltage indicator, ThT, is higher when the cell is more polarized, and the fluorescence of the potassium indicator, APG, is higher when the extracellular potassium concentration is higher. The fluorescence for voltage and potassium are highly correlated. (Right) Mean voltage and potassium fluorescence from the model. The quantities from the model show a similar pattern to those in the experiment. Note that the fluorescence data from the experiment are scaled differently than the model data (Color figure online)

We see that in experiments from Prindle et al. (2015) the average external potassium increases within the biofilm as the average voltage differential decreases. This property is integral to the system because it reflects how the signal is propagated within the biofilm. If a cell releases potassium, its voltage differential becomes more negative because the cell is losing a positive ion. A newly released potassium ion may enter a neighboring cell, but the voltage change from this uptake should not more than offset the voltage change in the biofilm caused by the ion leaving its previous cell. This property has not been adequately reflected in previous models, such as in Martinez-Corral et al. (2019). We implement the model from this paper, and we show a resulting plot of the data in Fig. 4. We see that the voltage and potassium are inversely related in the original form. However, when we modify the equation to use a Neuman flux boundary condition and a constant internal diffusion rate, the voltage and potassium’s relationship change. The modified model shows an increase in the voltage during a period when cells are releasing potassium, which appears to not reflect the physical constraints of the system. In Fig. 2, we see that our model correctly indicates that the voltage falls as the external potassium increases. Potassium and voltage are not strictly inversely related because the potassium diffuses out of the biofilm causing the potassium levels to drop before the rise in the voltage. We compare these quantities to data from Prindle et al. (2015) in Fig. 5 where the fluorescence indicating hyperpolarization and the fluorescence indicating external potassium rise and fall in relative synchronicity for both the experimental and the model data. The initial conditions used in the one-dimensional simulations are $G=30$ mM, $K=8$ mM, $G_{in}=20$ mM, $K_{in}=300$ mM, $K_{acclimated}=8$ mM, $V=-156$ mV, and $n=0.1$ throughout the biofilm and the initial width of 0.2 mm for the biofilm.

### Size at Oscillation Onset and Period Length

**Fig. 6:**
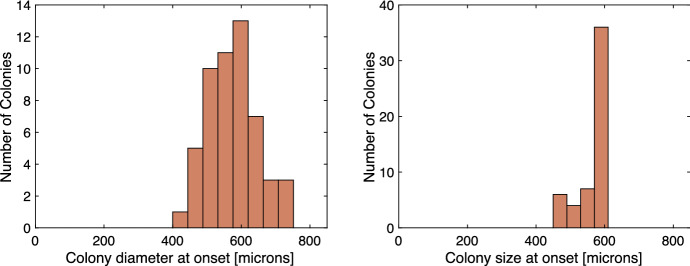
(Left) Experimental biofilm size at the onset of oscillations in 30 millimolar solution of glutamate, adapted from Jintao et al. (2015).^2^ The plot has 53 observations. The mean diameter at oscillation onset is near 600 $\mu$m. (Right) Onset size of oscillations for model biofilm under the same glutamate concentration. The size is calculated as twice the biofilm’s width when the mean voltage stops decreasing and starts increasing. We double the model’s width to better match the experimental measurement of diameter. The plot has 53 observations. The modeled biofilm has a mean onset size between 500 and 600 $\mu$m. The small variation seen in the model is due to randomly initiating the biofilm’s size near 70 microns at the start of the simulation (Color figure online)

The size at which the biofilm initiates its first oscillation depends on the nutrient availability and the cellular consumption rate of the glutamate within the biofilm. We fit the growth rate of the model so that the biofilm initiates its oscillatory behavior at a similar size to experiments with 30 millimolar glutamate solutions. The initial biofilm width in the simulations is 70 microns with the addition of a random uniform variable ranging from $\pm 25$ microns. In the simulation, the start of an oscillation is defined as the moment when the biofilm’s mean voltage stops decreasing and begins to increase. In Fig. 6, we double the reported width of the simulated biofilm to compare it to the experimental measurement, which reports the diameter of the biofilm instead of its radius. For some initializations, the modeled biofilm displays one unsustained oscillation at the start of the simulation. To remove these spurious oscillations from the model’s onset data, we do not include unsustained oscillations that occur in the simulation before the biofilm’s doubled width reaches at least 300 microns. In Fig. 6, we observe that in both the experiments and the model the mean size at which the biofilms begin to oscillate is near 500 microns. The experiments have a larger variability around the mean than the model, which is typical of the natural variability inherent in physical systems.^1^

**Fig. 7:**
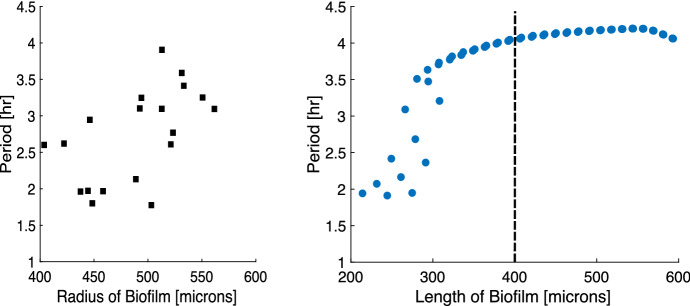
(Left) Experimental period of oscillation by biofilm size (radius) adapted from Martinez-Corral et al. (2018) with permission from Rosa Martinez-Corral. The oscillation period lengthens as the biofilm grows. (Right) Period of oscillation by biofilm size (width in 1D model) from the model from three initial biofilm widths. The size is calculated as the biofilm’s size when the mean voltage transitions from decreasing to increasing. To the right of the dashed line is model data from the same domain as the experimental data. To the left of the dashed line is model data that shows a lengthening of the oscillation period (Color figure online)

**Fig. 8:**
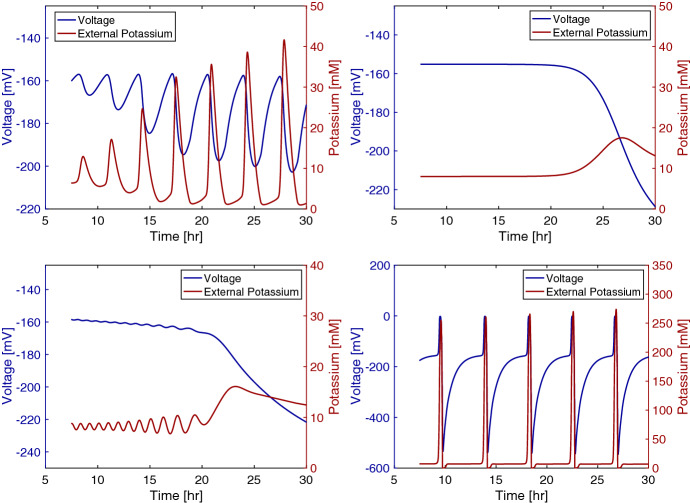
Mean voltage and potassium over time: (Top Left) from the original model, (Top Right) from a model with a constant Grow Mode, $M_{grow}\equiv 0.2$, (Bottom Left) from a model in which the cells do not adjust to the changing external potassium concentrations, $K_{acclimated}\equiv 0$ and $\delta _L=6$, (Bottom Right) from a model in which potassium does not leak in and out of the cell through the leak gates, see Eqs. (17) and (18) (Color figure online)

In both the model and the experiments, we see that the period of oscillation generally increases as the biofilm’s size increases, as shown in Fig. 7. The simulated width we report is the width of the one-dimensional model, which we compare with the experimental radius measurements. Though these measurements are different, we can still compare the general trend. The initial width of the biofilm in the model is 80, 90, and 100 microns. The simulated biofilm begins to oscillate at different widths depending on its initial width, which contributes to the variation seen in the model data. Some of the oscillations begin as only small changes in the voltage and growth rate, which can have shorter periods. In the model data, we see that most of the increase in period length occurs before the biofilm reaches 400 microns, after which the biofilm appears to converge to a longer period of oscillation in Fig. 7. This convergence may be the result of the varying activity levels of the bacteria. The depth of this participatory portion near the fluid interface may reach a limit as the biofilm grows larger while the center of the biofilm remains consistently hyperpolarized. We have not explored if a similar phenomenon occurs in experimental biofilms to test if the period of oscillation converges to a similar oscillation period.

In Fig. 8, we observe how the behavior of the model changes under a few important modifications: setting the Grow Mode, $M_{grow}$ to be constant, having the bacteria react to the absolute potassium concentration, and eliminating the leaking of potassium ions into the cells. In these comparisons, we initiate the biofilm at 150 microns and use the same initial conditions for the quantities that we used in the previous figures. The first modification we perform is setting the Grow Mode variable to be constant at $M_{grow} \equiv 0.2$. We chose this value so that the biofilm hyperpolarizes gradually as it grows. If we set $M_{grow}\equiv 1$, the biofilm’s growth rate is high enough that the biofilm hyperpolarizes at the start of the simulation. With $M_{grow} \equiv 0.2$, the model no longer oscillates, which suggests that the bacterial growth regulation is an important component of the oscillations. Next, we show how the model’s behavior changes if the cells no longer react to a change in potassium but to the absolute potassium level, a feature common to other models. In this case, we set $K_{acclimated}\equiv 0$ so that the bacteria cannot adapt to a change in potassium, and we lower $\delta _L$ to 6 mV/mM, or $10\%$ of its fitted value, to reduce the biofilm’s reactivity to the ion. If $\delta _L$ remains at its full value within the modified model, the biofilm may be too reactive to find an equilibrium before it begins to hyperpolarize. We see small oscillations occur in the voltage and potassium, which could a product of the initialization where the biofilm is initiated away from its equilibrium value. However, these oscillations do not achieve a similar magnitude to the oscillations from our original model, and eventually they are damped. It appears that the model biofilm’s ability to adjust to a changing potassium concentration improves its ability to oscillate like the experimental biofilm. Finally, we compare the oscillatory biofilm model to a model in which the potassium does not leak back into the bacteria. We, respectively, change Eqs. (2) and (6) to

17 $$\begin{aligned} \frac{\partial K}{\partial t}&= D_K\frac{\partial ^2K}{\partial x^2}+Fg_Kn^4(V-V_K)- \max (\gamma _KK(K_{max}-K_{in}),0), \end{aligned}$$

18 $$\begin{aligned} \frac{d K_{in}}{dt}&= -Fg_Kn^4(V-V_K) + \max (\gamma _KK(K_{max}-K_{in}),0)- \frac{\partial }{\partial x} (U K_{in}). \end{aligned}$$

Without potassium leaking back into the cell, the potassium and voltage spike together in what could be a feedback reaction. Then, both the voltage and potassium fall together. This relationship between voltage and potassium appears to not follow the physical constraints of the system because voltage typically falls as the cells release potassium. Our model’s addition of the leak gate term to the potassium equation improves its ability to represent the physical system.

Most of the variables used here were chosen because their combination produces results that were qualitatively similar to the experimental data we use for comparison. Their accuracy would likely improve from a closer quantitative study. However, quantitatively comparing the model to the data is often difficult because we do not have precise models that relate the fluorescence data from the experiments to the underlying voltage differential and potassium concentration. Here, we perform a sensitivity analysis to better understand the oscillations so that future work may focus on important parameters to address the quantitative accuracy of the model. We report the results in Table 2.

In the sensitivity analysis, we consider the magnitude of the potassium oscillations as a proxy for the oscillation’s strength. We begin the simulations at a biofilm width of 300 microns and run the simulation for a 20-hour time period. We then calculate the mean potassium value within the biofilm at each time point during the 20-hour period and find the difference between the highest and lowest values, which gives the magnitude of the potassium oscillations. We calculate the sensitivity by adding a 0.001 difference to each parameter and use a finite difference to approximate the derivative. Since each variable is not scaled equally, we also report the percent change in the magnitude of the potassium oscillations with respect to a $10\%$ change in each parameter. We calculate this percent change in the magnitude of the potassium oscillations through the following formula

$$\begin{aligned} \frac{\partial O}{\partial p} \times \frac{ 0.1 |p|}{O} \times 100\%,\end{aligned}$$

where *O* is the magnitude of the oscillations and *p* is the value of the parameter.

**Table 2:** Sensitivity of parameters used in simulation

Name	Approximate derivative of the magnitude of the potassium oscillation with respect to parameter	Percent change in the magnitude of the potassium oscillation with a $10\%$ change in parameter
$D_G$	$-33.2737$	$-2.3871\%$
$D_K$	$-37.8966$	$-4.5312\%$
$D_G^{fl}$	$-28.1194$	$-1.8559\%$
$D_K^{fl}$	$-1.7722$	$-1.1697\%$
$G_0$	$-1.5467$	$-6.1646\%$
$K_0$	0.5807	$0.6172\%$
$\delta _G$	0.6480	$0.8609\%$
$V_{th}$	3.1252	$62.2792\%$
$G_{max}$	3.2329	$8.5900\%$
*F*	6.8217	$5.0752\%$
$g_K$	0.1039	$2.4856\%$
$g_L$	$-8.4926$	$-1.3539\%$
$\gamma _K$	$-128.5507$	$-0.4270\%$
$B_L$	85.7137	$5.6936\%$
$\gamma _G$	34.1090	$5.0979\%$
$r_b$	106.5717	$1.4158\%$
$G_u$	$-0.4833$	$-1.1558\%$
$\eta _V$	$-0.6851$	$-1.8203\%$
$\gamma _V$	2.8506	$7.5740\%$
$V_{low}$	$-2.6697$	$-62.0683\%$
$\delta _{grow}$	313.3146	$0.3122\%$
$\eta _K$	0.4915	$1.9589\%$
$\alpha$	8.6587	$5.7516\%$
$\beta$	$-14.0939$	$-4.6810\%$
*m*	$-1.0573$	$-0.2809\%$
$G_l$	3.7702	$5.0088\%$
$V_{K0}$	$-0.2268$	$-11.4517\%$
$V_{L0}$	0.0966	$2.0028\%$
$\delta _K$	$-22.1445$	$-2.9419\%$
$\delta _L$	$-0.1369$	$-1.0909\%$

It appears that $V_{th}$, which affects the glutamate uptake, and $V_{low}$, which affects the growth rate, have the largest percent effect on the magnitude of the potassium oscillations. Raising $V_{th}$ increases the magnitude of the potassium oscillations by reducing the bacteria’s ability to uptake glutamate, increasing the bacteria’s propensity to hyperpolarize in order to uptake glutamate. Raising $V_{low}$ decreases the magnitude of potassium oscillations by reducing the bacteria’s growth rate and reducing their glutamate usage, thereby reducing the bacteria’s propensity to hyperpolarize to uptake glutamate. The oscillations are likely sensitive to $V_{th}$ and to $V_{low}$ because they are incorporated into an exponential function and hyperbolic tangent function, respectively, which can produce large derivatives.

It appears that $V_{K0}$, the reversal potential, has the next highest relative effect on the magnitude of the potassium oscillations. Raising $V_{K0}$ decreases the bacteria’s ability to hyperpolarize, and reduces the magnitude of oscillations. The potassium oscillations are also sensitive to the variables that relate to glutamate consumption, such as $G_{max}$, $\gamma _G$, and $G_l$, which play an important role in the cell’s starvation process.

The diffusion constants within the biofilm, $D_G$ and $D_K$, appear to play a role in the strength of the oscillations. In this model, we have assumed that the parameters are fractions of the diffusion constants in water. Though the percent influence that they have on the oscillations does not appear to be high enough to qualitatively affect the oscillations, future work should take a closer look at the physical motivation of these parameters.

The variables related to ThT and APG fluorescence are not reported in Table 2. The equations related to ThT and APG convert the voltage differential and potassium concentration to quantities that we can experimentally measure, but they do not affect the oscillations themselves.

Now that we have examined the properties of our one-dimensional model, we use this set of equations to create a two-dimensional model. The two-dimensional model allows us to explore the spatial complexity of the oscillations. We present this two-dimensional model in the following section.

## Two-Dimensional Model

The true benefit of the two-dimensional model is that we can directly model the behavior of a biofilm from an experiment by inputting the biofilm’s complex shape into the simulation. In this section, we introduce a two-dimensional model to represent the data from the experiments performed in Prindle et al. (2015). The flow cell in their experiment is about 3 mm long and 3 mm wide but only 5–7 microns in depth. The narrow depth only allows the biofilm to grow 5–7 cells deep, which confines the biofilm to a two-dimensional layer. This cross section allows the experimentalists to visualize properties within the biofilm that would typically not be visible from the exterior of a three-dimensional biofilm. By viewing a two-dimensional “slice” of a biofilm, we can track how the electrical impulse moves from the interior to the exterior of the biofilm.

We model the propagation of this signal using a two-dimensional model based on the continuum model developed in Merkey et al. (2009). This system uses a Stokes-flow approximation to solve for the fluid velocity through the flow cell. Let *u* and *v* be the $x-$ and $y-$directional fluid velocities, $\mu$ be the fluid viscosity, and *P* be the pressure field. Then, the reduced equations for the fluid flow are

$$\begin{aligned} \mu \nabla ^2 u= & {} \frac{\partial P}{\partial x},\\ \mu \nabla ^2 v= & {} \frac{\partial P}{\partial y},\\ \nabla ^2 P= & {} 0. \end{aligned}$$

The flow cell has a rectangular shape defined by an inlet on the left side, an outlet on the right side, and walls on the top and the bottom of the domain. The boundary condition for the fluid at the interior walls and at the biofilm boundary is a no-slip and no-penetration boundary condition where $u=0$, $v=0$, and $\nabla P \cdot \mathbf {n}=0$, where $\mathbf {n}$ in the outward normal. At the inlet, we use the boundary conditions $u= u_0$, $v = 0$, and $\partial u/\partial x=0$, where $u_0$ is the initial speed. At the outlet we approximate a far-field boundary with the conditions $\partial u/\partial x = 0$, $v=0$, and $P=0$.

We then solve for the concentrations of the diffusive quantities glutamate and potassium in both the fluid and the biofilm. In the fluid, these equations are

19 $$\begin{aligned}&D_G^{fl}\nabla ^2 G -\nabla \cdot (G\langle u,v \rangle ) = 0, \end{aligned}$$

20 $$\begin{aligned}&\quad D_K^{fl}\nabla ^2 K -\nabla \cdot (K\langle u,v \rangle ) = 0, \end{aligned}$$

with the boundary conditions $G=G_0$ and $K=K_0$ at the inlet, no-flux conditions at the interior walls in which $\nabla G\cdot \mathbf {n} = \nabla K\cdot \mathbf {n} = 0$, and far-field conditions at the outlet in which $\nabla G\cdot \mathbf {n} = \nabla K\cdot \mathbf {n} = 0$ where $\mathbf {n}$ represents the outward normal at the boundary. At the biofilm interface, the boundary conditions for *G* and *K* require that the concentrations and the fluxes are continuous across the interface, which lead to the following equations:

21 $$\begin{aligned} D_G \nabla G_{int}\cdot \mathbf {n}&= D_G^{fl} \nabla G_{ext}\cdot \mathbf {n}, \end{aligned}$$

22 $$\begin{aligned} G_{int}&= G_{ext}, \end{aligned}$$

23 $$\begin{aligned} D_K \nabla K_{int}\cdot \mathbf {n}&= D_K^{fl} \nabla K_{ext}\cdot \mathbf {n}, \end{aligned}$$

24 $$\begin{aligned} K_{int}&= K_{ext}, \end{aligned}$$

where $G_{int}$, $G_{ext}$, $K_{int}$, $K_{ext}$ refer to the glutamate and potassium concentrations inside and outside the biofilm at the interface, respectively, and $\mathbf {n}$ is the outward normal.

We solve the same equations as in the one-dimensional model for the diffusive quantities glutamate and potassium within the biofilm using Eqs. (1) and (2). We solve the non-diffusive quantities, or the cellular state variables, only within the biofilm and not in the surrounding fluid. For the cellular state variables, we use Eqs. (5–7) and (10–16) where the advection component of the equations uses the multidimensional biomass velocity and the multidimensional $\nabla$ operator. We solve these equations on a two-dimensional grid that covers the flow-cell domain.

In the two-dimensional model the growth equations, which replace Eqs. (8) and (9), use a potential function to approximate the viscous flow induced by the cellular growth throughout the biofilm

25 $$\begin{aligned} \varOmega= & {} \delta _{grow}\nabla ^2\left( M_{grow} \times G_{in} \right) , \end{aligned}$$

26 $$\begin{aligned} \mathbf {U}= & {} \nabla \varOmega , \end{aligned}$$

where $\mathbf {U}$ evaluated at the boundary of the biofilm gives the directional growth of the boundary. We track the biofilm growth and the moving biofilm-fluid interface using the level-set method. The level-set method was introduced in Osher and Sethian (1988) and discussed further in Sethian (1999), Osher and Fedkiw (2003), and references therein. Following work from Merkey et al. (2009), we use the zero level-set function of $\phi$ to track the biofilm interface, where $\phi$ solves the following equation:

27 $$\begin{aligned} \frac{\partial \phi }{\partial t} = \mathbf {U}\cdot \mathbf {n} ||\nabla \phi ||, \end{aligned}$$

where $\mathbf {n}$ is the outward normal evaluated at the points along the biofilm boundary where $\phi = 0$. The level-set method ensures that the boundary grows at the rate determined by Eq. (26). After implementing this set of equations within a two-dimensional simulation, we compare the model results to experiments.

### Comparing the Two-Dimensional Model to Experiments

In this paper, we initialize a biofilm to be the same shape as a biofilm from the experiments in Prindle et al. (2015) with the fluid inlet on the right. The fluid velocity at the inlet is parabolic across the chamber with a maximum velocity of 90 millimeters per hour in the center and 0 at the top and bottom walls, which is close to the fluid velocity used in experiments. The initial conditions for the two-dimensional simulations are $G=30$ mM, $K=8$ mM, $G_{in}=20$ mM, $K_{in}=300$ mM, $K_{acclimated}=8$ mM, $V=-160$ mV, and $n=0.1$. The simulations demonstrate that the bacteria located farther from the fluid interface become stressed due to nutrient limitation, and they release potassium to hyperpolarize. This voltage change travels from the interior of the biofilm near the flow-cell wall to the fluid interface. In this process, the whole biofilm hyperpolarizes from the inside out.

**Fig. 9:**
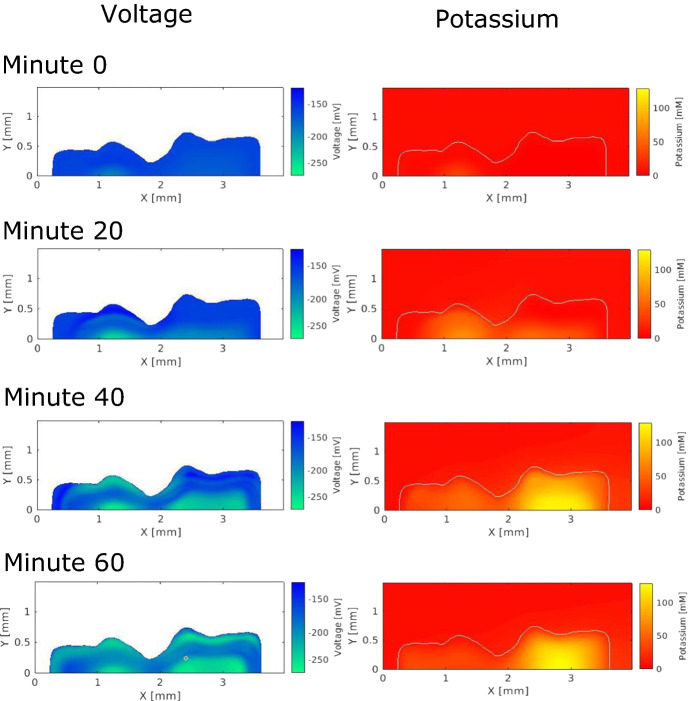
Voltage and potassium within a two-dimensional biofilm simulation during one oscillation. The white background in the voltage plots indicates no data and the white line in the potassium plots indicates the edge of the biofilm (Color figure online)

The results of this model are shown in Fig. 9 in which we can see the hyperpolarization and the potassium wave spread throughout the simulated biofilm. The videos of these quantities from the model can be found in the supplementary material. The voltage plots show that the hyperpolarization moves from the center of the biomass to the fluid interface. The center of the biofilm as well as the peripheral regions become highly hyperpolarized at minute 60. The middle region only hyperpolarizes a small amount initially, but as can be seen in the accompanying videos, hyperpolarizes a large amount later in the oscillation. The delayed hyperpolarization of the middle region could be a result of the $M_{grow}$ variable reflecting metabolic adaptation. The cells near the interface grow and have a high glutamate consumption rate, which leads to a high level of stress when they experience the potassium wave. The high stress could lead the peripheral cells to quickly hyperpolarize. The middle region consumes glutamate more slowly, which could delay and mitigate their stress response.

The potassium plots in Fig. 9 show how the oscillations initiate and propagate. The left side of the biofilm initiates its hyperpolarization first. However, the region on the right experiences a higher-amplitude potassium pulse. The magnitude of the right side’s pulse could be due in part to the thickness of the right region or that the region is downstream and experiences slightly lower environmental glutamate levels. We visually estimate the potassium wave speed to be around 20–50 microns per minute. This wave speed is similar to those seen in experiments such as in Martinez-Corral et al. (2019) which provides data from one experiment in which we visually estimate the experimental wave speed to be near 10 microns per minute. The period of the oscillations in the simulation is around three to four hours. This period is a bit longer than the period in the reference experiment, in which the period appears to be around two to three hours. However, the experimental period can take on a range of values, as shown in Fig. 7.

**Fig. 10:**
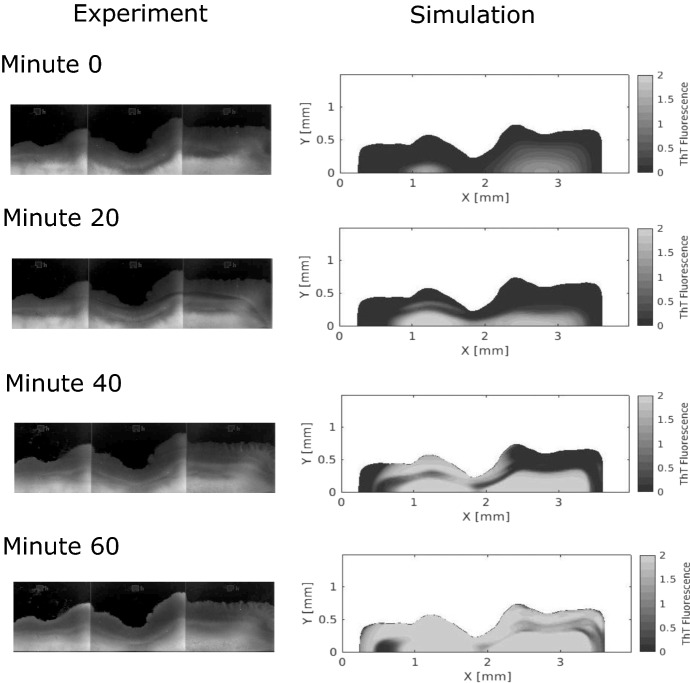
ThT fluorescence from an experiment compared to a simulation during one oscillation. The imaged region in the experimental data has roughly a length of 3 mm and a width of 1 mm. The two vertical lines in the experimental fluorescence data correspond to slight changes in the flow-cell depth. The black background in the experimental plots indicates no data, and the white background of the simulation plots indicates no data (Color figure online)

**Fig. 11:**
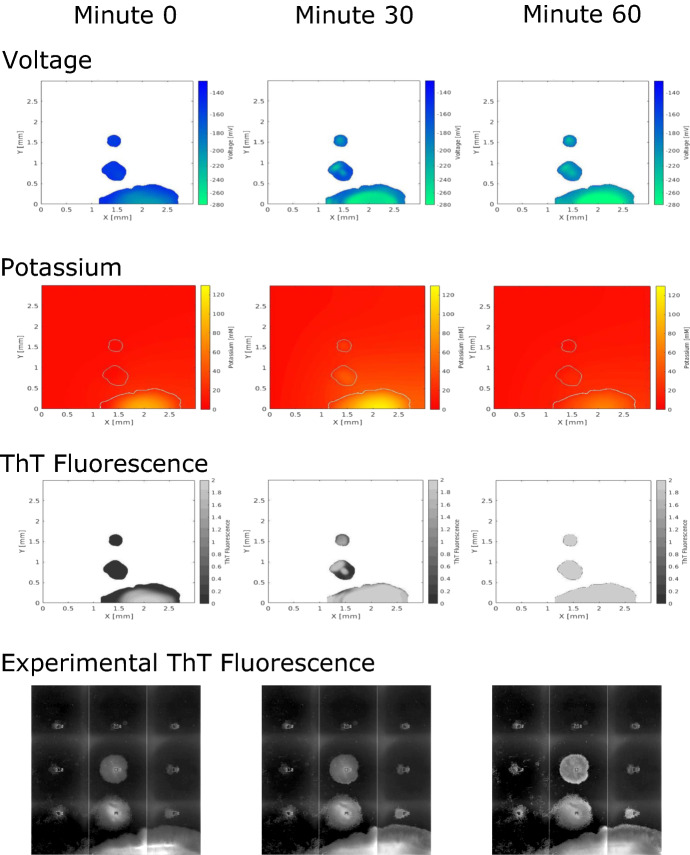
Simulated voltage, potassium, and ThT fluorescence of multiple biofilms within a flow cell. We compare the data to experimental ThT fluorescence with the same initial shape. The depolarization spreads between separated biofilms as potassium diffuses within the fluid. The depolarization wave begins in the large biofilm on the bottom of the flow cell and spreads to the other biofilms. The imaged region in the experimental data has a length of 3 mm and a width of 3 mm. The vertical line in the experimental fluorescence of the biofilm on the bottom wall corresponds to a slight change in the flow-cell depth. White space in the model images indicate no data and the white lines in the potassium images indicate the boundaries of the biofilms (Color figure online)

Potassium is not confined to the interior of the biofilm, and it diffuses out of the biofilm and flows downstream. The release of potassium affects spatially separated bacteria within the flow cell. We take a closer look at the effects of potassium within the fluid in the following section in which we model multiple interacting biofilms within the same flow cell.

We also model ThT fluorescence within the biofilm and compare these distributions to experimental data in Fig. 10. The video of this quantity from the model can be found in the supplementary material. The modeled ThT fluorescence does not match the experiment at every spatial point, but the hyperpolarization pattern is qualitatively similar. In both the experiment and the model, the fluorescence is high near the wall of the flow cell, far from the fluid interface. We also see the high fluorescence wave travel from the interior to the exterior, which indicates the spread of hyperpolarization.

Another interesting feature of the model is the spatial banding that appears as the biofilm hyperpolarizes. The middle of the biofilm is slower to become highly fluorescent than the periphery, even though the potassium wave travels through the middle to reach the fluid interface. As suggested earlier, this effect could be a result of the $M_{grow}$ variable, which limits the growth and glutamate consumption of this middle region. There appears to be some amount of fluorescent banding in the experimental data as well. At minute 60, the periphery of the experimental biofilm has a higher fluorescence than the center of the biofilm. We would like to study this effect further.

The model we develop here can capture many important spatial features of the oscillations, which enables further study of these behaviors. Now that we have compared the simulated behavior of a single biofilm to the experimental data, we demonstrate the full utility of this two-dimensional model in modeling an entire flow cell with interacting biofilms in the following section.

### Modeling Interacting Biofilms within a Flow Cell

Many of the experiments performed on this biofilm system are implemented within flow cells with multiple biofilms. One-dimensional models are not able to capture the interactions between multiple biofilms within a flow cell. The flow cells have multiple cell traps that catch planktonic bacteria as they flow through the chamber and provide the seeds for distinct biofilms. These biofilms grow and influence their neighbors both by consuming glutamate and by releasing potassium. We demonstrate that the simulation developed here is capable of modeling interacting biofilms.

We use our two-dimensional simulation to model multiple biofilms in a flow cell that is 3 millimeters by 3 millimeters. The fluid velocity at the inlet is parabolic with a maximum velocity of 10 millimeters per hour in the center and 0 next to the walls of the flow cell. The velocity at the inlet was chosen so that each biofilm oscillates at its initial size, and it is lower than the experimental velocity to ensure that the biofilms synchronize in the model. The initial shapes of the biofilms are taken from the comparison experiment. We calculate the voltage, potassium, and ThT fluorescence from the experimental setup in Prindle et al. (2015), and we show the results in Fig. 11. The videos of these quantities from the model can be found in the supplementary material. We simulate three biofilms in the flow cell: a large biofilm on the bottom and two smaller biofilms above the larger one. The large biofilm begins to oscillate first because its size causes the bacteria at its center to become nutrient starved before the bacteria in the other biofilms. We see that the oscillations in voltage, potassium, and ThT of each biofilm in the model are synchronized since they are likely driven by the hyperpolarization of the largest biofilm at the bottom wall. In the experimental ThT fluorescence in Fig. 11, we also see a synchronization in oscillation that is likely driven by the oscillations of the larger biofilm at the bottom of the flow cell. We would like to explore the properties of this synchronization in future work.

## Conclusion

In this paper, we introduced and discussed a new model for electrical communication in *B. subtilis*. We updated the propagation mechanism to depend on the change in potassium level instead of the absolute potassium level, which incorporates bacteria’s ability to adjust to environmental changes and, in our experience, produced oscillations similar to those seen in experiments under a larger range of parameters. We also updated the boundary condition at the biofilm interface and the potassium leak mechanism so that the potassium and the voltage oscillations are more synchronized.

Using this new model, we explored the relationship between voltage, growth, and potassium, showing that the voltage and the growth follow a similar pattern to each other while the voltage differential and the extracellular potassium are closer to inversely related. We also examined the diffusive properties of the system including how the biofilm’s size relates to the initiation and the period of its oscillations.

We then adapted this model into a two-dimensional system using the level-set method to track the boundary. The ability to simulate a two-dimensional biofilm allows us to copy a biofilm’s shape from an experiment and closely compare the model to the physical system. We examined the voltage, the potassium, and the ThT fluorescence within the model. We are also able to examine the interactions between multiple biofilms within a flow cell and observe how separated biofilms synchronize by releasing potassium into the flow. The model demonstrates synchronization patterns similar to those in experiments.

This model can enable researchers to computationally explore this biofilm system in connection with their experiments. This model can be used to test hypotheses about the biofilms such as those relating to the growth speed, the effect of potassium on cells, and how collective oscillations can arise, or fail to arise, based on the individual behavior of the cells. The greatest benefit of this model is that we can compare the model’s results by directly copying the shapes of experimental biofilms, which allows researchers to study the two-dimensional properties of the system. For example, researchers can study the wave speed of the potassium signal through spatially segregated cells of differing phenotypes. We can also explore properties such as the emergence of oscillations within a single biofilm and the synchronization between separated biofilms within a single flow cell. Understanding the spatial properties of the system can help scientists find methods to control the biofilm’s growth and dispersal in more realistic environments, potentially leading to new ways to treat biofilm growth and any associated infections.

In future research, we would like to further explore cellular metabolism and the synchronization of oscillations initializing from different regions within a biofilm. First, we would like to understand how a rise in external potassium disrupts cellular metabolism. We believe that this study could provide a better understanding of the cellular mechanisms that create biofilm-wide oscillations. In particular, studying metabolism could give us further insight into the depolarization and recovery process of a cell and how it regulates growth using a mechanism such as the Grow Mode presented here. We would then like to explore how metabolism and changes in growth rates either lead to synchronicity within the biofilm or allow for regional divergence in oscillations within large biofilms.

This oscillatory behavior of *Bacillus subtilis* involves many complex processes, and we are just beginning to put the pieces together. Understanding the components of the oscillations could inspire new methods to influence and even control certain biofilms’ behavior, such as the attachment and growth, using potassium signaling. These new control methods would not rely on harsh treatments such as antibiotics but on adjusting the biofilm’s environmental conditions to influence its oscillatory behavior.

## Supplementary Information

Below is the link to the electronic supplementary material.

Supplementary material 1 (mp4 329 KB)

Supplementary material 2 (mp4 258 KB)

Supplementary material 3 (mp4 196 KB)

Supplementary material 4 (mp4 421 KB)

Supplementary material 5 (mp4 586 KB)

Supplementary material 6 (mp4 303 KB)

## Footnotes

1 Adapted by permission from Springer Nature Customer Service Centre GmbH: Springer Nature, Nature, Ion channels enable electrical communication in bacterial communities, Prindle et al. Copyright 2015 Macmillan Publishers Limited. (2015), https://www.nature.com.

2 Adapted by permission from Springer Nature Customer Service Centre GmbH: Springer Nature, Nature, Metabolic co-dependence gives rise to collective oscillations within biofilms, Jintao Liu et Al. Copyright 2015 Macmillan Publishers Limited. (2015), https://www.nature.com.
