# Correction to: A Two-Dimensional Model of Potassium Signaling and Oscillatory Growth in a Biofilm

**DOI:** 10.1007/s11538-022-01003-9

**Published:** 2022-02-18

**Authors:** Noah Ford, Garth Fisher, Arthur Prindle, David Chopp

**Affiliations:** 1 Engineering Sciences and Applied Mathematics Department, Northwestern University, Evanston, IL 60208 USA; 2 Department of Biochemistry and Molecular Genetics, Feinberg School of Medicine, Northwestern University, Chicago, IL 60611 USA; 3 Center for Synthetic Biology, Northwestern University, Evanston, IL 60208 USA; 4 Department of Chemical and Biological Engineering, Northwestern University, Evanston, IL 60208 USA

## Correction to: Bulletin of Mathematical Biology (2021) 83:60 10.1007/s11538-021-00887-3

The article “A Two-Dimensional Model of Potassium Signaling and Oscillatory Growth in a Biofilm” written by “Noah Ford, Garth Fisher, Arthur Prindle, David Chopp”, was originally published Online First without Open Access. After publication in volume 83, issue 5, the author decided to opt for Open Choice and to make the article an Open Access publication. Therefore, the copyright of the article has been changed to © The Authors 2021 and the article is forthwith distributed under the terms of the Creative Commons Attribution 4.0 International License, which permits use, sharing, adaptation, distribution and reproduction in any medium or format, as long as you give appropriate credit to the original author(s) and the source, provide a link to the Creative Commons licence, and indicate if changes were made. The images or other third party material in this article are included in the article’s Creative Commons licence, unless indicated otherwise in a credit line to the material. If material is not included in the article’s Creative Commons licence and your intended use is not permitted by statutory regulation or exceeds the permitted use, you will need to obtain permission directly from the copyright holder. To view a copy of this licence, visit https://creativecommons.org/licenses/by/4.0

